# GPR110 ligands reduce chronic optic tract gliosis and visual deficit following repetitive mild traumatic brain injury in mice

**DOI:** 10.1186/s12974-021-02195-y

**Published:** 2021-07-17

**Authors:** Huazhen Chen, Karl Kevala, Elma Aflaki, Juan Marugan, Hee-Yong Kim

**Affiliations:** 1 Laboratory of Molecular Signaling, NIAAA, NIH, 5625 Fishers Lane, Rockville, MD 20852 USA; 2 Center for Neuroscience and Regenerative Medicine, Henry M. Jackson Foundation, Bethesda, MD 20817 USA; 3 Division of Pre-Clinical Innovation, NCATS, NIH, Rockville, MD 20817 USA

**Keywords:** Synaptamide, A8 (4Z,7Z,10Z,13Z,16Z,19Z)-N-(2-hydroxy-2-methylpropyl) docosa-4,7,10,13,16,19-hexaenamide (dimethylsynaptamide), repetitive CHIMERA, GPR110, Histopathology, Optic tract, Visual deficit, VEP, mTBI

## Abstract

**Background:**

Repetitive mild traumatic brain injury (mTBI) can result in chronic visual dysfunction. G-protein receptor 110 (GPR110, ADGRF1) is the target receptor of *N*-docosahexaenoylethanolamine (synaptamide) mediating the anti-neuroinflammatory function of synaptamide. In this study, we evaluated the effect of an endogenous and a synthetic ligand of GPR110, synaptamide and (4Z,7Z,10Z,13Z,16Z,19Z)-N-(2-hydroxy-2-methylpropyl) docosa-4,7,10,13,16,19-hexaenamide (dimethylsynaptamide, A8), on the mTBI-induced long-term optic tract histopathology and visual dysfunction using Closed-Head Impact Model of Engineered Rotational Acceleration (CHIMERA), a clinically relevant model of mTBI.

**Methods:**

The brain injury in wild-type (WT) and GPR110 knockout (KO) mice was induced by CHIMERA applied daily for 3 days, and GPR110 ligands were intraperitoneally injected immediately following each impact. The expression of GPR110 and proinflammatory mediator tumor necrosis factor (TNF) in the brain was measured by using real-time quantitative reverse transcription polymerase chain reaction (qRT-PCR) in an acute phase. Chronic inflammatory responses in the optic tract and visual dysfunction were assessed by immunostaining for Iba-1 and GFAP and visual evoked potential (VEP), respectively. The effect of GPR110 ligands *in vitro* was evaluated by the cyclic adenosine monophosphate (cAMP) production in primary microglia isolated from adult WT or KO mouse brains.

**Results:**

CHIMERA injury acutely upregulated the GPR110 and TNF gene level in mouse brain. Repetitive CHIMERA (rCHIMERA) increased the GFAP and Iba-1 immunostaining of glia cells and silver staining of degenerating axons in the optic tract with significant reduction of N1 amplitude of visual evoked potential at up to 3.5 months after injury. Both GPR110 ligands dose- and GPR110-dependently increased cAMP in cultured primary microglia with A8, a ligand with improved stability, being more effective than synaptamide. Intraperitoneal injection of A8 at 1 mg/kg or synaptamide at 5 mg/kg significantly reduced the acute expression of TNF mRNA in the brain and ameliorated chronic optic tract microgliosis, astrogliosis, and axonal degeneration as well as visual deficit caused by injury in WT but not in GPR110 KO mice.

**Conclusion:**

Our data demonstrate that ligand-induced activation of the GPR110/cAMP system upregulated after injury ameliorates the long-term optic tract histopathology and visual impairment caused by rCHIMERA. Based on the anti-inflammatory nature of GPR110 activation, we suggest that GPR110 ligands may have therapeutic potential for chronic visual dysfunction associated with mTBI.

**Supplementary Information:**

The online version contains supplementary material available at 10.1186/s12974-021-02195-y.

## Introduction

The white matter of brain, mainly composed of myelinated axons that relay a coordinating communication of grey matter of brain, is known to be susceptible to the impact of the acceleration/deceleration forces [1–3]. The optic tract is particularly vulnerable, and the disruption of the visual process can be detected even in mild TBI (mTBI) [4–6], especially in repeated mild close head brain injury such as blast-related injury [7]. According to the report from DVBIC (the Defense and Veterans Brain Injury Center), approximately 74% of the service members in Iraq and Afghanistan wars diagnosed with TBI since 2000 has experienced visual impairments that adversely affect the cognitive performance and quality of individual life. We and others have reported that repetitive mTBI in animal models produces profound neuropathological changes in the optic tract and visual deficit at acute and chronic phases [6, 8–11]. Although the visual deficit is an obvious confounding factor for motor, sensory, cognitive, and neuropsychiatric symptoms of TBI, there has been little effort for developing effective strategy to improve the TBI-linked visual deficit.

Synaptamide (*N*-docosahexaenoylethanolamin) is a metabolite of docosahexaenoic acid, an omega-3 fatty acid enriched in the brain [12]. Synaptamide is an endogenous ligand of GPR110 (ADGRF1) that belongs to the adhesion G-protein coupled receptor (GPCR) group VI family [13]. By binding to the GPCR-autoproteolysis-inducing domain of GPR110, synaptamide triggers the downstream cAMP/PKA/CREB signaling pathway [13, 14] and stimulates neurite outgrowth and synaptogenesis in mouse cortical neuron and induces neuronal differentiation of mouse neural stem cells at nanomolar concentrations [13, 15]. In an animal model of neuroinflammation induced by lipopolysaccharide (LPS), GPR110-dependent anti-inflammatory effects of synaptamide have also been observed [16, 17], suggesting GPR110 as a potential therapeutic target for neuroinflammation. In addition, (4Z,7Z,10Z,13Z,16Z,19Z)-N-(2-hydroxy-2-methylpropyl) docosa-4,7,10,13,16,19-hexaenamide (dimethylsynaptamide, A8), a chemical analogue of synaptamide with improved stability, was recently shown to be an effective GPR110 ligand that can activate cAMP signaling for axon regeneration [18]. Closed-head impact model of engineered rotational acceleration (CHIMERA) is a suitable animal model to study the long-term pathophysiology of TBI [9, 19], and repetitive CHIMERA (rCHIMERA) can produce significant visual deficit along with the glia activation and axon degeneration in the optic tract [11]. In this study, we investigated the therapeutic potential of GPR110 ligands in repetitive mTBI based on the optic tract histopathology and visual deficit induced by rCHIMERA and anti-inflammatory effects of GPR110/cAMP signaling.

## Materials and methods

### Experimental animals

Mice were housed in the animal facility of the National Institute on Alcohol Abuse and Alcoholism (NIAAA) with free access to standard food and water under a 12-h light–dark cycle. GPR110 heterozygous mice on C57BL/6J genetic background were generated by the Knockout Mouse Project (KOMP) Repository (MMRRC_046507-UCD). Heterozygous GPR110 male and female mice were bred to produce GPR110 knockout offspring. The WT littermates were used as controls. Adult male and female mice at an age of 2–4 months were used for all experiments. GPR110 KO mice developed normally and showed no visible abnormal phototype at this age. All animal experiments were conducted according to the NIH guidelines for the health and use of laboratory animals (LMS-HK-13).

### rCHIMERA injury and treatments

**Scheme 1:**
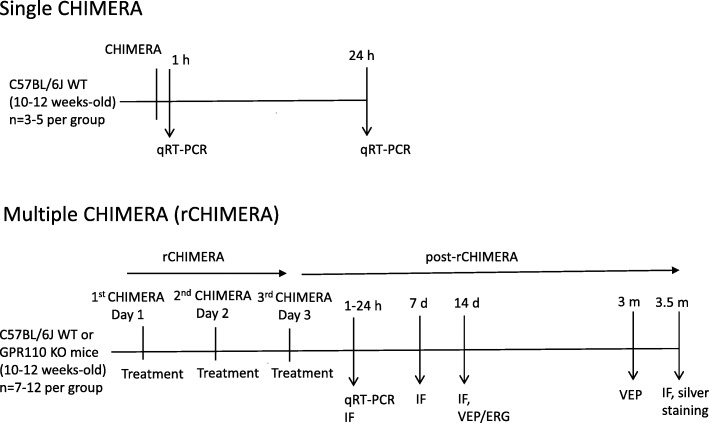
The experimental scheme

The mild closed traumatic brain injury was induced by rCHIMERA (rCHI) as previously described [9]. Briefly, mice were anesthetized with 5% isoflurane in oxygen (1 L/min) for 3 min and placed in a supine position on the CHIMERA apparatus with the head lying flat on the hole of the base plate. An impact of 0.55 J of energy was delivered to the mouse head by a computer-controlled pneumatic air pressure. An impact was given daily for three consecutive days at a 24-h interval, and immediately following each impact, mice were intraperitoneally injected with vehicle, synaptamide, oleoylethanolamide (OEA), or A8 that was synthesized as previously reported [18]. The mice were returned to the home cage and allowed to recover, and the samples were collected according to the experimental timeline shown in Scheme 1. WT and GPR110 KO mice were randomly divided into four groups: (1) Sham, (2) rCHI + V, (3) rCHI + SYN, and (4) rCHI + A8. The solution of synaptamide and A8 for the intraperitoneal (i.p.) injection was freshly prepared from the DMSO stock by diluting with mixture of *N,N*-dimethylacetamide (DMAC, Sigma-Aldrich, St. Louis, MO, USA, cat# 271012-1L): Solutol H15 (Sigma-Aldrich, St. Louis, MO, USA, cat# 42966 ): 1 × PBS (1:1:2). The investigator who performed the experiment was blinded with respect to the compound identity for treatments until all data analyses were completed.

### Quantitative real-time reverse transcription polymerase chain reaction (RT-PCR)

Mice were anesthetized with isoflurane and transcardially perfused with 0.1 M phosphate buffer (pH 7.4). The cerebral cortex was rapidly dissected, and total RNA was extracted by Trizol reagent (Thermo Fisher Scientific, cat# 15596026, MA, USA), reverse transcribed to cDNA with High Capacity cDNA Reverse Transcription kit (Applied Biosystem, cat# 4368814, CA, USA). Quantitative RT-PCR was performed using TaqMan™ probes (Thermo Fisher Scientific) run on the QuantStudio 3 Real-Time PCR system (Applied Biosystems by Thermo Fisher Scientific). cDNA samples were reacted with the TaqMan™ probe for GPR110 (Assay ID: Mm00505409_m1, cat# 4331182) or TNF (Assay ID: Mm00443258_m1, cat# 4331182) using TaqMan™ Fast Advanced Master Mix (Thermo Fisher Scientific, cat# 4444557) in the presence of the TaqMan™ probe for HPRT (hypoxanthine guanine phosphoribosyl transferase) (Assay ID: Mm00446968_m1, cat# 4448490) which was used as an internal control. Data were analyzed using comparative Ct method. The relative mRNA level of target gene normalization to HPRT was calculated as 2^-ΔΔCt^ value.

### Immunofluorescence and silver staining

The animals were deeply anesthetized with isoflurane and transcardially perfused with 0.1 M phosphate buffer (pH 7.4) and fixed with 4% paraformaldehyde. The brains were carefully removed and post-fixed in 4% paraformaldehyde solution for overnight at 4 °C and subsequently transferred into 30% sucrose solution at 4 °C. The brains were embedded with O.C.T. compound medium (VWR, PA, USA, cat# 25608-930) and frozen on dry ice and stored at – 80 °C. Coronal sections (25 μm thickness) were sliced by Leica Cryostat (Leica Biosystems Inc., IL, USA) and stored in cryoprotective solution at – 20 °C. Three sections per mouse (approximately bregma 1.68 to − 2.2 mm) were selected for staining.

The immunofluorescence double staining for Iba-1 and GFAP was conducted according to the procedure previously described [9]. The sections were incubated with Iba-1 (Wako Chemicals, VA, USA, cat# 019-19741) and GFAP antibodies (Novus Biologicals, CO, USA, cat# NBP1-05198) at 4 °C for overnight and Alexa fluor 488-conjugated F (ab’)2 fragment goat anti-rabbit IgG and sheep anti-chicken IgG (Jackson Immuno-Research labs, PA, USA, cat# 703-035-155) at RT for 1 h. After washing, the sections were mounted on the slides and covered with mounting medium containing DAPI (Vector laboratories, CA, USA, cat# H-1500). Immunofluorescence images were captured by an Olympus 1 × 81 microscopic system. The quantification of GFAP and Iba-1 expression was performed by measuring the fluorescence intensity (per μm^2^) using Metamorph software (Molecular Devices Inc., CA, USA).

The silver staining was performed using FD NeuroSilver™ Kit (FD NeuroTechnologies, Inc, MD, USA, cat# PK301A) according to the manufacturer's protocol. The 20×-magnified images of the optic tract were acquired in bright field with an Olympus 1 × 81 microscopic system. The staining intensity of images was quantified using Image J (NIH, MD, USA).

### Visual evoked potential and electroretinogram

The visual evoked potential (VEP) and electroretinogram (ERG) were recorded with the Epsion Visual Electrophysiology System (Diagnosys, LLC, MA, USA). All procedures were performed under dim red light after the mice were acclimated in the room at least for 1 h. Mice were anesthetized with an intraperitoneal injection of ketamine and xylazine cocktail solution (100 mg/kg ketamine + 10 mg/kg xylazine). Each pupil of the mouse was dilated with a drop of 2.5% phenylephrine hydrochloride solution before being placed on a heated platform of color dome. A reference electrode was placed in the lower lip of the mouse and a ground electrode placed on the tail. For VEP measurements, the active electrode was subcutaneously inserted in the middle of the two ears. Both eyes were stimulated by the flash stimuli of white light (6500 K) at an intensity of 3.0 cd s/m^2^ repeatedly with each set including 100 sweeps. Three sets of readings were recorded and averaged to obtain the amplitude and latency (implicit time) of the N1 component (the first negative peak: P1–N1) as described earlier [11]. For ERG recording, a drop of topical petrolatum ophthalmic ointment was applied to the corneal surface of one eye on which a gold wire electrode was placed with the other eye covered. A light-adapted (photopic) protocol was used for ERG measurement as described earlier [20]. After testing, the mice were transferred to the home cage and placed on a heat pad to recover. The investigator was blinded regarding the identity of the experimental groups to prevent bias.

### Determination of the brain synaptamide and A8 level

Synaptamide and A8 were analyzed by reversed phase liquid chromatography coupled to high-resolution tandem mass spectrometry using a Thermo Scientific Q-Exactive mass spectrometer. Mice were intraperitoneally injected with a mixture of d4-synaptamide and A8. At 1, 2, and 24 h after injection, mice were perfused with 1× PBS, and the cerebral cortex was collected and homogenized in 500 μL of water/methanol (1:1) mixture containing 2 μM URB597 (a fatty acid amide hydrolase inhibitor) and 50 μg/mL butyl hydroxytoluene (BHT) (Sigma-Aldrich, St. Louis, MO, USA, cat# W218405). Protein concentration of the homogenate was determined by the bicinchoninic acid protein assay kit (Thermo Fisher Scientific, Waltham, MA, USA, cat# 23225). A mixture of deuterated internal standards of d_4_-anandamide and d_6_-A8 was added to 300 μL of homogenate, which was then brought to BHT-methanol/water (7:3) and centrifuged for 20 min at 4 °C. Supernatants were loaded onto a Strata-X polymeric C18 reverse-phase SPE cartridge (33 μm, 30 mg/mL, Phenomenex, Torrance, CA, USA) that was wetted with BHT-methanol and equilibrated with water. After washing with water, samples were eluted with 2.5 mL BHT-methanol into glass tubes, dried under N2, and resuspended in a small volume of BHT-methanol. Separation was made on an Eclipse C18 HPLC column (1.8 μm, 2.1 mm × 50 mm, Agilent Technologies, Santa Clara, CA, USA) using a tertiary gradient consisting of water (A), methanol (B), and acetonitrile (Avantor, Radnor Township, PA, USA) (C), with all solvents containing 0.01% acetic acid (Thermo Scientific). After pre-equilibration of column with A/B (60%/40%), 5 μL extract was injected, and the solvent composition was linearly changed to A/B/C (36.3%/15%/48.7%) in 5 min, followed by a linear gradient to A/B/C (13.5%/68.4%/18.1%) over 22 min. The mass transitions of 376.3 to 66.085, 400.3 to 72.081, 352.3 to 66.085, and 406.4 to 78.118 were used to detect d_4_-synaptamide, analog 8, d_4_-anandamide, and d_6_-A8, respectively. Quantitation of d_4_-synaptamide and A8 was made using d_4_-anandamide and d_6_-A8 as the respective internal standard. Results were normalized to protein amount and presented as fmol per microgram protein.

### Determination of cAMP in primary microglia

The brain tissue from GPR110-WT or KO mice at 4 months of age were dissociated into single cells using the Adult Brain Dissociation Kit (Miltenyi Biotec, Gaithersburg, MD, USA, cat#130-107-677) according to the manufacturer’s instruction. To isolate the microglia, the single-cell suspension was incubated with CD11b Microbeads for 30 min at 4 °C. The cells were resuspended in MACS buffer and passed through the LS column (Miltenyi Biotech). The column was washed 4 times with MACS buffer and then magnetically labeled CD11b-positive cells were flushed out of the columns twice using MACS buffer. Enriched CD11b-positive microglia cells were pelleted by centrifuging at 300 × g at 4 °C for 10 min and resuspended in DMEM media supplemented with 10% fetal bovine serum (FBS). Isolated CD11b-positive microglia cells were cultured in poly-D-lysin coated 384-well white/clear bottom plate for 7 days by changing 50% of DMEM media supplemented with 10% FBS every other day. On the day of the cAMP assay, media was changed to the stimulation media consisting of phenol red-free DMEM supplemented with 0.5 mM IBMX (Tocris, Minneapolis, MN, USA), a phosphodiesterase inhibitor. Microglia cells were treated with different concentrations of synaptamide or A8 complexed with 0.05% fatty acid-free BSA (Sigma-Aldrich) in the presence of 40 μM vitamin E for 10 min. Separately, microglia cells were treated with 10 μM Forskolin (Tocris, cas# 66575-29-9) for 10 min. The cAMP production was measured by a homogenous time-resolved fluorescence assay with a cAMP Gs Dynamic Kit (Cis Bio, Bedford, MA, USA, cat#62AM4PEB) using the FlexStaion 3 device (Molecular devices)

### Statistical analysis

The data are presented as mean ± standard error of the mean (SEM). Statistical analyses were performed using GraphPad Prism (version 6.04, GraphPad Software Inc.) for one-way or two-way analysis of variance (ANOVA) followed by the Tukey’s post hoc test for multiple comparisons, and *p* < 0.05 was considered statistically significant.

## Results

### CHIMERA induces GPR110 gene expression in the brain

**Fig. 1:**
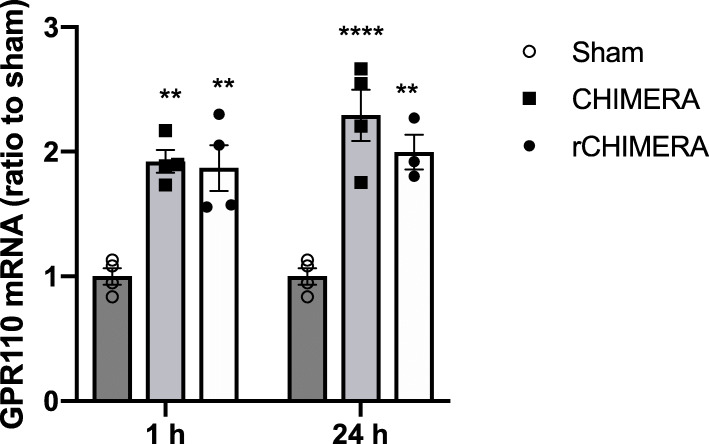
Induction of GPR110 mRNA by single or multiple CHIMERA. The GPR110 mRNA in brain measured by RT-PCR using TaqMan probes shows upregulation at 1 h and 24 h after single (CHIMERA) or repeated CHIMERA applied daily for 3 days at a 24-h interval (rCHIMERA). The data are expressed as mean ± SEM (n = 3–4) and are representative of three independent experiments. **p < 0.05; ****p < 0.0001 vs. Sham

To investigate the possible role of GPR110 activation in improving TBI outcome, we first examined the GPR110 mRNA expression in adult mouse brain using quantitative RT-PCR at 1 and 24 h after single or multiple CHIMERA given daily for three consecutive days. Both single and multiple CHIMERA significantly upregulated GPR110 gene level at 1 and 24 h after injury compared to the sham group (F = 38.53, *p* < 0.0001, n = 3-4/group, Fig. 1), indicating that *gpr*110 expression responds quickly to the brain injury.

### GPR110 ligands dose-dependently stimulate cAMP production in primary microglia

**Fig. 2:**
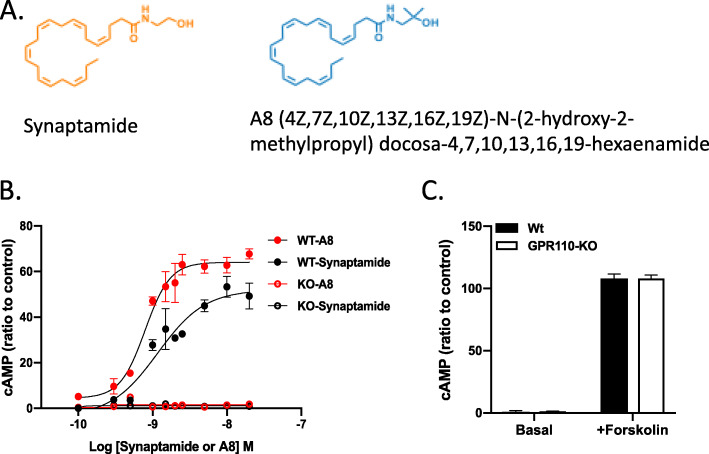
Dose-dependent effect of GPR110 ligands on cAMP production in mouse primary microglia cells. **A** The chemical structure of GPR110 ligands, synaptamide and A8. **B** The concentration-dependent production of cAMP in primary microglia stimulated by GPR110 ligands. The EC50 values of synaptamide and A8 are 1.27 and 0.79 nM. Microglia isolated from adult GPR110-WT and KO mice were treated with varying concentrations of synaptamide or A8 for 10 min. The fold change data are presented as mean ± SEM from a representative experiment out of three independent experiments performed in triplicates. **C** The cAMP production in primary microglia after stimulation with forskolin. No difference was found in the cAMP production from WT and KO microglia. The microglia cells were treated with 10 μM forskolin for 10 min and cAMP production was measured in triplicate. The data is representative of three independent experiments

A methylated analogue of synaptamide, (4Z,7Z,10Z,13Z,16Z,19Z)-N-(2-hydroxy-2-methylpropyl)docosa-4,7,10,13,16,19-hexaenamide (A8, NCGC00248435) (Fig. 2A), was previously shown to be resistant to hydrolysis by fatty acid amide hydrolase (FAAH) and to produce cAMP more effectively than synaptamide in cortical neurons [18]. Since rCHIMERA induces microglia activation, we examined the capability of this ligand to stimulate cAMP production in primary microglia (Fig. 2B) where GPR110 was shown to be expressed [17]. As shown in cortical neurons [18], A8 dose-dependently increased microglial production of cAMP more effectively than synaptamide with EC_50_ of 0.79 nM. This increase was GPR110-dependent as the microglia from GPR110 KO mice did not respond to A8 while forskolin raised the cAMP level in both preparations (Fig. 2C).

### Determination of treatment dose and ligand stability *in vivo*

**Fig. 3:**
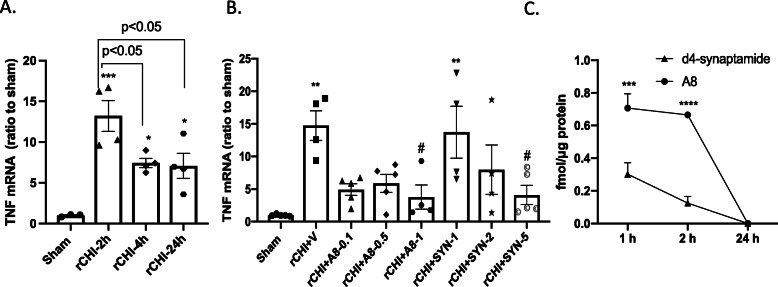
Dose-dependent effect of synaptamide and A8 on TNF mRNA expression and their stability *in vivo*. **A** The mRNA level of TNF in the mouse cortex quantified by qRT-PCR using TaqMan probes at 2, 4, and 24 h after rCHIMERA. The TNF mRNA level from injured mice was upregulated at all time points compared with Sham control (***p < 0.001, *p < 0.05 vs. Sham) with the most pronounced increase occurring at 2 h after the last injury (*p < 0.05 vs. rCHI-4 h or rCHI-24 h). The data are expressed as mean ± SEM (n = 4). Each dot symbol represents an individual animal within each group. **B** Dose-dependent effects of GPR110 ligands in cortical TNF mRNA level evaluated at 2 h after the last injury. Mice were intraperitoneally (i.p.) injected with varying doses of synaptamide (SYN, 1, 2, and 5 mg/kg) or A8 (0.1, 0.5, 1 mg/kg) immediately following each injury. The increase in the mRNA level of TNF after rCHI (rCHI + V, **p < 0.001 vs. Sham) was significantly suppressed by A8 at 1 mg/kg and synaptamide at 5 mg/kg *(#p < 0.05* vs. rCHI + V). The data are expressed as mean ± SEM (n = 4–5). Each dot symbol represents an individual animal within each group. **C** The time course of A8 and d_4_-synaptamide detected in the mouse brain. A8 (1 mg/kg) and d_4_-synaptamide (5 mg/kg) intraperitoneally injected were detected by tandem mass spectrometry. The A8 level in mouse cortex is significantly higher than synaptamide at 1 h and 2 h after intraperitoneal injection. The data are expressed as mean ± SEM (n = 3). **p < 0.001, ***p < 0.001

To determine the proper dose of GPR110 ligands for *in vivo* treatment, we examined their effects on the expression of an inflammation marker TNF after rCHIMERA. We chose the time point at 2 h after rCHIMERA because the maximum expression of TNF mRNA occurred at this time although it remained elevated throughout the duration of experiment up to 24 h after injury (F = 12.61, *p* < 0.001, *n* = 3–4/group, Fig. 3A). Based on the previous finding that synaptamide at 5 mg/kg significantly suppressed LPS-induced neuroinflammation [16, 17] as well as the effectiveness of A8 in microglia (Fig. 2C), we examined the dose range of 1–5 mg/kg for synaptamide and 0.1–1 mg/kg for A8 (Fig. 3B). We found that the brain mRNA level of TNF was significantly upregulated at 2 h after injury (F = 5.1, *p* < 0.001, 14.7 ± 2.27-fold, *p* < 0.001 vs. Sham). Intraperitoneal administration of A8 or synaptamide immediately following each CHIMERA dose-dependently suppressed the TNF expression. Compared with the vehicle-treated injured group (rCHI + V), synaptamide at 5 mg/kg or A8 at 1 mg/kg significantly suppressed TNF expression (*p* < 0.05 and *p* < 0.05 vs. rCHI + V for 1 mg/kg A8 and 5 mg/kg synaptamide, respectively). Synaptamide at 2 mg/kg or A8 at 0.1 and 0.5 mg/kg also showed the trend to reduce the TNF mRNA expression although the reduction was not statistically significant. The time–course of synaptamide and A8 in the brain was examined by injecting d_4_-synaptamide at 5 mg/kg and A8 at 1 mg/kg (Fig. 3C). Both d_4_-synaptamide and A8 were detected in the brain cortex obtained after transcardiac perfusion, but their level was less than 1% of the initial amounts injected at all time points. At 1 and 2 h after injection, the stable analogue A8 was found in the brain cortex at a significantly higher level (0.71 ± 0.09 and 0.67 ± 0.02 fmol/μg protein at 1 and 2 h, respectively) than synaptamide (0.3 ± 0.07 and 0.13 ± 0.04 fmol/μg protein at 1 and 2 h, respectively), even though the injected dose of A8 was 5-fold less compared with synaptamide. Both compounds were no longer detectable in the brain cortex after 24 h, indicating rapid metabolism and clearance of these compounds. Considering these results, we selected 5 mg/kg of synaptamide and 1 mg/kg of A8 for treatment for *in vivo* experiments.

### Synaptamide and A8 suppress rCHIMERA-induced gliosis in a GPR110-dependent manner

**Fig. 4:**
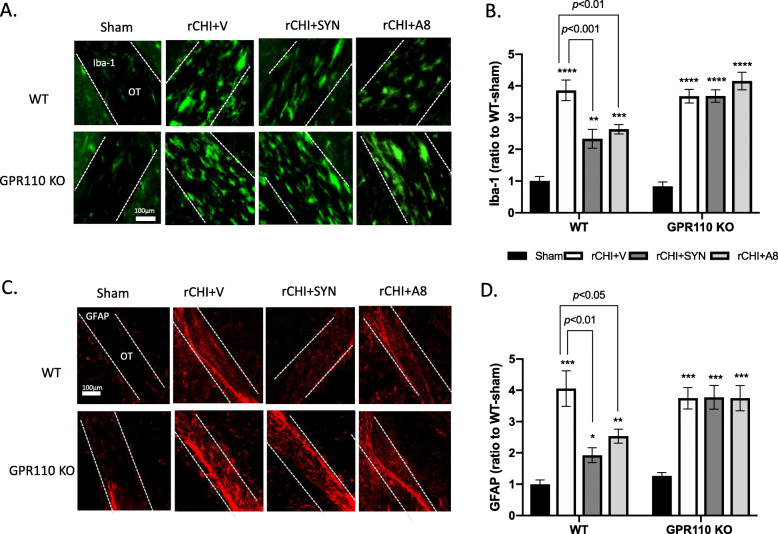
GPR110-dependent amelioration of rCHIMERA-induced glia cell activation by synaptamide and A8. **A**, **C** Representative immune-fluorescence micrographic images of Iba-1 (**A**) and GFAP (**C**) in the optic tract from WT and GPR110 KO mice at 1 week after injury (rCHI). WT and GPR110 KO mice were injected with A8 (1 mg/kg, i.p.) or synaptamide (5 mg/kg, i.p.) after each CHIMERA, and brains were collected for immunostaining at 7 days after the last injury. **B**, **D** Quantitative analysis showing significant suppression of Iba-1 (**B**) and GFAP expression (**D**) by the treatment with synaptamide or A8 compared to the vehicle-treated group (rCHI + V) in WT but not in GPR110 KO mice after rCHIMERA. The optic tract region (OT) is outlined with dashed lines. The data are expressed as mean ± SEM (n = 3). *p < 0.05, **p < 0.01, ***p < 0.001 vs. Sham-WT

**Fig. 5:**
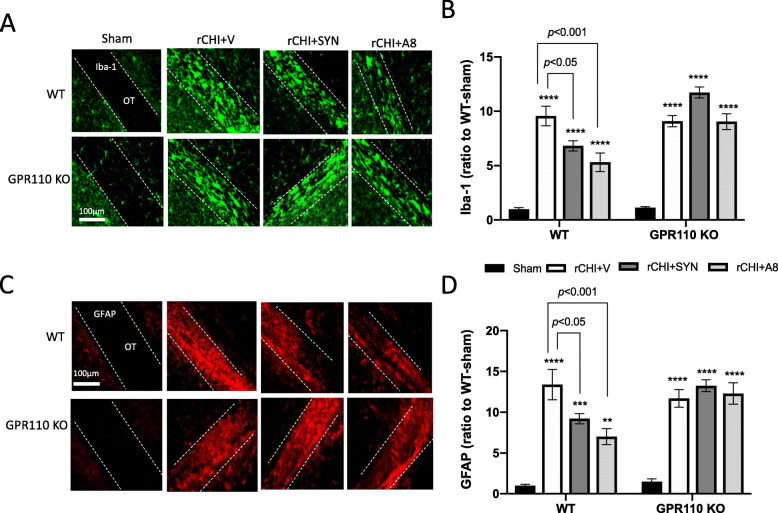
GPR110-dependent attenuation of rCHIMERA-induced chronic gliosis by synaptamide and A8. **A**, **C**. Representative immune-fluorescence micrographic images of Iba-1 (**A**) and GFAP (**C**) in the optic tract from WT and GPR110 KO mice at 3.5 months after injury (rCHI). WT and GPR110 KO mice were injected with A8 (1 mg/kg, i.p.) or synaptamide (5 mg/kg, i.p.) after each CHIMERA, and brains were collected for immunostaining at 3.5 months after the last injury. **B**, **D** Quantitative analysis showing significant suppression of Iba-1 (**B**) and GFAP expression (**D**) by the treatment with synaptamide or A8 compared to the vehicle-treated group (rCHI + V) in WT but not in GPR110 KO mice after rCHIMERA. The optic tract region (OT) is outlined with dashed lines. The data are expressed as mean ± SEM (n = 3). **p < 0.01, ****p < 0.0001 vs. Sham-WT

A number of studies have been reported that repetitive mild TBI results in microglia and astrocyte activation in white matter [21–24]. We examined the effect of GPR110 ligands on the expression of GFAP (an astrocyte marker) and Iba-1 (a microglia marker) in the optic tract (OT) at 24 h (Fig. S1) and 7 days after injury by rCHIMERA (Fig. 4). At 24 h after injury, synaptamide significantly suppressed the Iba-1 (F = 43.63, *p* < 0.0001 vs. rCHI + V) and GFAP expression in OT (F = 14.5, *p* < 0.0001vs. rCHI + V) but *N*-oleoylethanolamine (OEA), an inactive lipid control, showed no effect, indicating that the suppression of glia cell activation was a specific effect of synaptamide (Fig. S1). Similarly, significant increases in the expression of Iba-1 (Fig. 4A, B) and GFAP (Fig. 4C, D) were observed in the OT from both WT and GPR110 KO mice at 7 days after injury (Iba-1: F = 34.11, *p* < 0.0001, GFAP: F = 15.38, *p* < 0.0001 vs. Sham-WT). Treating WT injured mice with synaptamide or A8 significantly reduced both Iba-1(rCHI + SYN vs. rCHI + V, *p* < 0.001; rCHI + A8 vs. rCHI + V, *p* < 0.01) and GFAP expression (rCHI + SYN vs. rCHI + V, *p* < 0.01; rCHI + A8 vs. rCHI + V, *p* < 0.05); however, this effect was not observed in GPR110 KO injured mice (Fig. 4B and D). A8 and synaptamide also produced similar effects, reducing glia activation in the corpus callosum at 7 days after injury (Fig. S2). To determine the long-term effects, we examined the expression of Iba-1 and GFAP in the OT at 3.5 months after injury (Fig. 5). Compared with the injured group with vehicle treatment (rCHI + V), synaptamide or A8 treatment significantly suppressed Iba-1 (rCHI + SYN vs. rCHI + V, *p* < 0.05; rCHI + A8 vs. rCHI + V, *p* < 0.001) and GFAP expression (rCHI + SYN vs. rCHI + V, *p* < 0.05; rCHI + A8 vs. rCHI + V, *p* < 0.001) in the OT of injured WT mice; however, injured GPR110 KO mice did not respond to the treatments (Fig. 5B and D). These results indicate that synaptamide and A8 inhibit gliosis in the optic tract in a GPR110-dependent manner.

### Synaptamide and A8 attenuate rCHIMERA-induced axonal damage in a GPR110-dependent manner

**Fig. 6:**
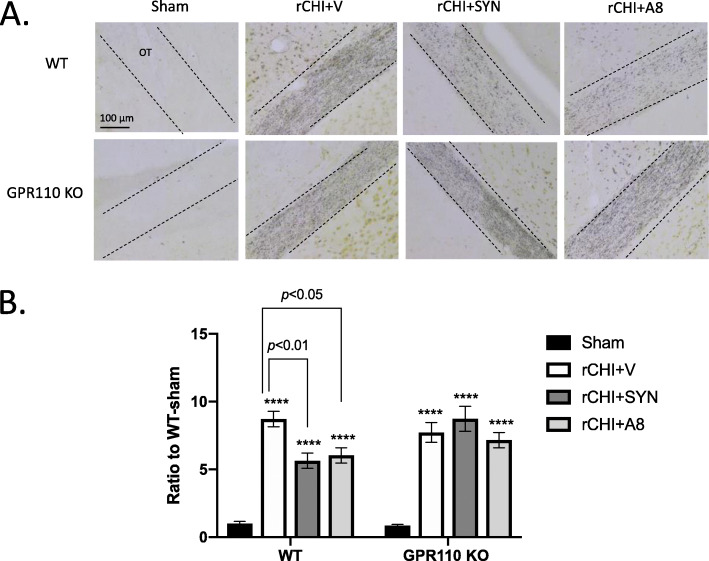
GPR110-dependent decrease in chronic axonal damage after treatment with synaptamide or A8. **A** Representative micrographic images of silver-stained axons in the optic tract from WT and GPR110 KO mice. WT and GPR110 KO mice were injected with synaptamide (5 mg/kg, i.p.) or A8 (1 mg/kg, i.p.) after each CHIMERA, and brains were collected for silver staining at 3.5 months after the last injury. **B** Quantitative analysis showing significant suppression of silver staining in the optic tract by the treatment with synaptamide or A8 compared to the vehicle-treated group (rCHI + V) in WT but not in GPR110 KO mice after rCHIMERA. The optic tract region (OT) is outlined with dashed lines. The data are expressed as mean ± SEM (n = 4). ***p < 0.001, ****p < 0.0001 vs. Sham-WT

It is well established that degenerating axons have a high affinity for silver ions [25]. Using silver staining, we examined the axonal damage in the OT from WT and KO mice at 3.5 months after injury. The pronounced increase in silver staining was observed in the OT from both rCHIMERA-injured WT and GPR110 KO mice (F = 44.48, *p* < 0.0001 vs. Sham). The silver staining intensity of OT from the injured mice treated with synaptamide or A8 was significantly reduced compared with the injured group treated with vehicle only (rCHI + SYN vs. rCHI + V, *p* < 0.01; rCHI + A8 vs. rCHI + V, *p* < 0.05). However, no such effect was observed in GPR110 KO mice after treatment with synaptamide or A8 (Fig. 6).

### GPR110 activation improved rCHIMERA-induced visual deficit

**Fig. 7:**
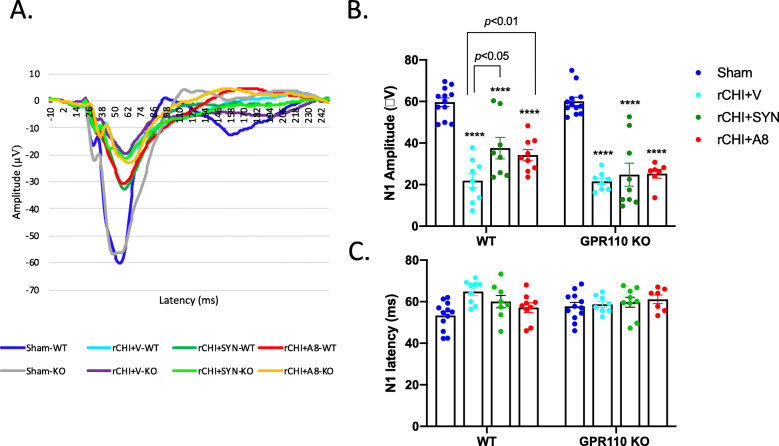
GPR110-dependent improvement of visual function impaired by rCHIMERA by synaptamide and A8. **A** Average trace of VEP evaluated at 3 months post injury. Full-field flash VEP was elicited at a constant intensity of 3.0 cd·s/m^2^ with the active electrode subcutaneously inserted in the middle of the two ears. **B**, **C** Quantitative analysis of the VEP response. N1 amplitude (**B**) and latency (**C**) indicates that treatment with synaptamide or A8 increased the N1 amplitude in WT but not in GPR110 KO injured mice without affecting N1 latency. No significant difference in the N1 amplitude or latency in sham animals was observed between two genotypes. The data are expressed as mean ± SEM (n = 7–12). Each dot symbol represents each animal per group. ***p < 0.001, ****p < 0.0001 vs. Sham-WT

Since rCHIMERA-induced upregulation of gliosis and axonal damage observed in the OT may be associated with visual dysfunction, we evaluated the visual evoke potential (VEP) along with the electroretinogram (ERG). At 2 weeks after injury, rCHIMERA significantly decreased N1 amplitude of VEP compared with the Sham group regardless of the genotype (F = 43.77, *p* < 0.0001; *p* < 0.001 vs. Sham for WT; *p* < 0.001vs. Sham for KO). The treatment with the GPR110 ligand A8 ameliorated this reduction in WT (*p* < 0.05 vs. rCHI + V) but not in GPR110 KO mice (Fig. S3A, B). Neither rCHIMERA nor GPR110 ligand affected the ERG as indicated by the unaltered amplitude and latency of a and b waves measured at 2 weeks after injury (Fig. S3C-E). A prolonged impact of rCHIMERA was also observed as the N1 amplitude was significantly reduced compared with the Sham group regardless of the genotype (F = 60.74, *p* < 0.0001; WT-rCHI + V: 21.88 ± 3.37 vs. Sham-WT: 59.61 ± 2.1, p < 0.01; KO-rCHI + V: 21.50 ± 1.57 vs. Sham-KO: 60.02 ± 1.98, p < 0.001) at 3 months post injury (Fig. 7). The N1 amplitude was significantly improved after the intraperitoneal injection of synaptamide (37.55 ± 5.20 μV, *p* < 0.05 vs. rCHI + V) or A8 (34.16 ± 2.65 μV, *p* < 0.01vs. rCHI + V) compared to the vehicle treatment after injury (21.88 ± 3.37 μV) in WT but not in GPR110 KO mice (Fig. 7A, B). The N1 latency was not significantly altered either by rCHIMERA or treatments with GPR110 ligands (Fig. 7C).

## Discussion

Numerous preclinical studies have searched for effective intervention for the long-lasting complications of repetitive mild traumatic brain injury. However, there are few successful candidates that can be translated into the clinic [26, 27]. In this study, we explored the therapeutic potential of GPR110 ligands, synaptamide and A8, in a clinically relevant mouse model of close head injury based on the optic tract neuropathology and visual dysfunction as the injury outcome. We found that these compounds produce GPR110-dependent amelioration of chronic neuropathology and vision deficit caused by rCHIMERA.

GPR110 is highly expressed in the neural stem cells, kidney, and developing brain but GPR110 expression is markedly diminished in the adult brain (Lee et al, 2016). In some cancer conditions such as prostate, liver, and breast cancer, upregulated expression of GPR110 has been reported [28–30]. Significant upregulation of GPR110 has also been demonstrated in adult neural tissues in response to injury [18] or LPS stimulation [17]. Likewise, the GPR110 gene level in adult mouse brains rapidly increased after single or multiple CHIMERA in our closed head injury model (Fig. 1).

G protein-coupled receptors (GPCRs) have been the targets for potential therapeutic agents in diverse fields [31, 32]. Many adhesion GPCRs are involved in cellular adhesion and signaling in immunology and neurology as well as developmental biology [33, 34]. GPR110 is the target receptor of synaptamide [13] that ameliorates LPS-induced neuroinflammation through the cAMP/PKA/CREB singling pathway *in vivo* and in cultured microglia where GPR110 is expressed [16, 17]. Recently, synaptamide has been shown to reduce inflammation and cognitive impairment in animal models of neuroinflammation and TBI [35, 36]. In LPS-induced neuroinflammation, synaptamide was shown to reduce the expression of proinflammatory cytokines/chemokines without affecting anti-inflammatory/pro-resolving cytokines [16]. Although the role of rapid induction of GPR110 after injury is not clear, the anti-inflammatory nature of GPR110 signaling may help attenuate uncontrolled inflammatory signals as part of the neuroprotective responses to injuries.

Neuroinflammation is an important mechanism underlying the pathogenesis of traumatic brain injury [37, 38], while microglia are potent immune effector cells producing and releasing proinflammatory and cytotoxic mediators in response to brain injury [23] Therefore, the GPR110-dependent microglial production of cAMP (Fig. 2), a well-established regulator of immune responses [39], is likely an important contributing mechanism to the effectiveness of GPR110 ligands on rCHIMERA-induced optic tract gliosis (Figs. 4 and 5, S1-S2) and visual dysfunction observed in this study (Fig. 7, S3). In addition, GPR110 ligands may have activated GPR110/cAMP signaling in other cellular components such as neurons and suppressed axonal damage (Fig. 6), also contributing to the improved injury outcome.

A8, a chemical analogue of synaptamide with improved stability, has been recently described as a better ligand to GPR110 compared with the endogenous ligand synaptamide [18]. Indeed, A8 was more effective for GPR110-dependent cAMP production in microglia (Fig. 2) and suppression of the inflammatory signal after brain injury caused by rCHIMERA (Fig. 3). Both A8 and synaptamide were detected in the brain after intraperitoneal injection indicating that they passed the brain–blood barrier and were delivered to the brain. The detected level of A8 was significantly higher compared to synaptamide although 5-fold less A8 was injected than synaptamide, confirming improved *in vivo* stability of A8. Enhanced *in vivo* stability and biological effectiveness of A8 suggest that A8 may have better translational potential than synaptamide.

Persistent gliosis caused by repetitive mild TBI is often associated with a functional deficit in animals and humans [22, 40–42]. The rCHIMERA-induced chronic gliosis and axonal degeneration in the optic tract [11, 19] are accompanied by visual impairment [11]. The vulnerability of the optic tract to mild TBI can be utilized for the evaluation of drug candidates for therapeutic potential in mild repetitive TBI. Such strategy led to the current demonstration of anti-inflammatory GPR110 ligands as effective agents in ameliorating the chronic optic tract histopathology and visual dysfunction. The positive effect of GPR110 ligands observed in the optic tract may occur similarly in other cerebral white matter tracts including the corpus callosum, internal capsule, and corticospinal tracts that are known to be disrupted significantly after TBI [43]. Consequently, these GPR110 ligands may find further applications to other brain functions impaired by mild repetitive TBI, particularly memory and executive function that require intact white matter tracts.

## Conclusions

Our results demonstrate that synaptamide and A8 attenuate optic tract histopathology and visual impairment caused by rCHIMERA by activating the GPR110/cAMP system that is upregulated after injury. This study provides new insight for the translational potential of targeting GPR110 using its ligands for improving the chronic outcome after repeated mild TBI.

## Supplementary Information

**Additional file 1: Figure S1.** Synaptamide-specific suppression of glia cell activation in optic tract (OT) at one day post-rCHIMERA. A. Representative micrographic images of Iba-1 and GFAP immunofluorescence. WT mice were injected with synaptamide or oleoylethanolamine (OEA) at 5 mg/kg (i.p.) after each CHIMERA, and brains were collected for immunostaining at 1 day after the last injury. B, C. Quantitative analysis of Iba-1 (B) and GFAP expression (B), showing that synaptamide suppressed Iba-1 and GFAP expression induced by rCHIMEA while oleoylethanolamine (OEA) had no effect. The synaptamide treatment without injury did not affect the GFAP and Iba-1 expression in the brain. The data are expressed as mean ± SEM (n=3). *p<0.05, **p<0.01, ****p<0.001 vs. Sham. **Figure S2.** GPR110-dependent inhibition of glia cell activation in corpus callosum by synaptamide and A8 at 7 days after injury. A, C. Representative micrographic images of Iba-1 (A) and GFAP (C) immunofluorescence in the corpus callosum (CC) from WT and GPR110 KO mice at 1 week after injury (rCHI). WT and GPR110 KO mice were injected with synaptamide (5 mg/kg, i.p.) or A8 (1 mg/kg, i.p.) after each CHIMERA, and brains were collected for immunostaining at 7 days after the last injury. B, D. Quantitative analysis showing significant suppression of Iba-1 (B) and GFAP expression (D) by the treatment with synaptamide or A8 compared to the vehicle-treated group (rCHI + V) in WT but not in GPR110 KO mice after rCHIMERA. The corpus callosum region (CC) is outlined with dashed lines. The data are expressed as mean ± SEM (n=3). *p<0.05, **p<0.01, ***p<0.001 vs. Sham-WT. **Figure S3.** Increases in N1 amplitude of VEP by A8 at 2 weeks after rCHIMERA. A: Average traces of VEP evaluated at 2 weeks post injury. Full-field flash VEP was elicited at a constant intensity of 3.0 cd·s/m^2^ with the active electrode subcutaneously inserted in the middle of the two ears. B, Quantitative analysis of the N1 amplitude and latency showing that A8 increased the N1 amplitude in WT but not in GPR110 KO injured mice without affecting N1 latency. No significant difference in the N1 amplitude or latency in sham animals was observed between two genotypes. The data are expressed as mean ± SEM (n=8-10). Each dot symbol represents each animal per group. ***p<0.001, ****p<0.0001 vs. Sham-WT. C. Average tracts of ERG at 2 weeks post injury obtained using a light-adapted (photopic) protocol. D, E. Quantitative analysis of a and b amplitude and latency showing that A8 or injury did not change a and b amplitude and latency for both WT and GPR110 KO mice. No significant difference in these ERG parameters was observed between two genotypes.

## Data Availability

The datasets used and/or analyzed during the current study are available from the corresponding author on reasonable request.

## Abbreviations

A8: (4Z,7Z,10Z,13Z,16Z,19Z)-N-(2-hydroxy-2-methylpropyl) docosa-4,7,10,13,16,19-hexaenamide
ANOVA: Analysis of variance
cAMP: Cyclic adenosine monophosphate
CHIMERA: Closed-Head Impact Model of Engineered Rotational Acceleration
ERG: Electroretinogram
FBS: Fetal bovine serum
GPCR: G-protein coupled receptor
GPR110: G-protein receptor 110
KO: Knockout
LPS: Lipopolysaccharide
mTBI: Mild traumatic brain injury
qRT-PCR: Quantitative reverse transcription polymerase chain reaction
rCHIMERA: Repetitive CHIMERA
SEM: Standard error of the mean
Synaptamide: *N*-Docosahexaenoylethanolamine
TNF: Tumor necrosis factor
VEP: Visual evoked potential
WT: Wild-type
