# Supplementary material for: GPR110 ligands reduce chronic optic tract gliosis and visual deficit following repetitive mild traumatic brain injury in mice
Source: J Neuroinflammation. 2021 Jul 17;18:157. doi: 10.1186/s12974-021-02195-y (PMC8286622; doi:10.1186/s12974-021-02195-y)
Supplement: Supplementary file 1 — Additional file 1: Figure S1. Synaptamide-specific suppression of glia cell activation in optic tract (OT) at one day post-rCHIMERA. A. Representative micrographic images of Iba-1 and GFAP immunofluorescence. WT mice were injected with synaptamide or oleoylethanolamine (OEA) at 5 mg/kg (i.p.) after each CHIMERA, and brains were collected for immunostaining at 1 day after the last injury. B, C. Quantitative analysis of Iba-1 (B) and GFAP expression (B), showing that synaptamide suppressed Iba-1 and GFAP expression induced by rCHIMEA while oleoylethanolamine (OEA) had no effect. The synaptamide treatment without injury did not affect the GFAP and Iba-1 expression in the brain. The data are expressed as mean ± SEM (n=3). *p<0.05, **p<0.01, ****p<0.001 vs. Sham. Figure S2. GPR110-dependent inhibition of glia cell activation in corpus callosum by synaptamide and A8 at 7 days after injury. A, C. Representative micrographic images of Iba-1 (A) and GFAP (C) immunofluorescence in the corpus callosum (CC) from WT and GPR110 KO mice at 1 week after injury (rCHI). WT and GPR110 KO mice were injected with synaptamide (5 mg/kg, i.p.) or A8 (1 mg/kg, i.p.) after each CHIMERA, and brains were collected for immunostaining at 7 days after the last injury. B, D. Quantitative analysis showing significant suppression of Iba-1 (B) and GFAP expression (D) by the treatment with synaptamide or A8 compared to the vehicle-treated group (rCHI + V) in WT but not in GPR110 KO mice after rCHIMERA. The corpus callosum region (CC) is outlined with dashed lines. The data are expressed as mean ± SEM (n=3). *p<0.05, **p<0.01, ***p<0.001 vs. Sham-WT. Figure S3. Increases in N1 amplitude of VEP by A8 at 2 weeks after rCHIMERA. A: Average traces of VEP evaluated at 2 weeks post injury. Full-field flash VEP was elicited at a constant intensity of 3.0 cd·s/m2 with the active electrode subcutaneously inserted in the middle of the two ears. B, Quantitative analysis of the N1 amplitude and latency showing tha [file 12974_2021_2195_MOESM1_ESM.docx]

**Fig. S1. Synaptamide-specific suppression of glia cell activation in optic tract (OT) at one day post-rCHIMERA**

A. Representative micrographic images of Iba-1 and GFAP immunofluorescence. WT mice were injected with syanptamide or oleoylethanolamine (OEA) at 5 mg/kg (i.p.) after each CHIMERA, and brains were collected for immunostaining at 1 day after the last injury.

B, C. Quantitative analysis of Iba-1 (B) and GFAP expression (B), showing that synaptamide suppressed Iba-1 and GFAP expression induced by rCHIMEA while oleoylethanolamine (OEA) had no effect. The synaptamide treatment without injury did not affect the GFAP and Iba-1 expression in the brain. The data are expressed as mean ± SEM (n=3). **p<0.05*, ***p<0.01*, *****p<0.001* vs. Sham.

**Fig. S2. GPR110-dependent inhibition of glia cell activation in corpus callosum by synaptamide and A8 at 7 days after injury**

A, C. Representative micrographic images of Iba-1 (A) and GFAP (C) immunofluorescence in the corpus callosum (CC) from WT and GPR110 KO mice at 1 week after injury (rCHI). WT and GPR110 KO mice were injected with synaptamide (5 mg/kg, i.p.) or A8 (1 mg/kg, i.p.) after each CHIMERA, and brains were collected for immunostaining at 7 days after the last injury.

B, D. Quantitative analysis showing significant suppression of Iba-1 (B) and GFAP expression (D) by the treatment with synaptamide or A8 compared to the vehicle-treated group (rCHI+V) in WT but not in GPR110 KO mice after rCHIMERA.

The corpus callosum region (CC) is outlined with dashed lines. The data are expressed as mean ± SEM (n=3). **p<0.05*, ***p<0.01*, ****p<0.001* vs. Sham-WT.

**Fig S3. Increases in N1 amplitude of VEP by A8 at 2 weeks after rCHIMERA**

A: Average traces of VEP evaluated at 2 weeks post injury. Full-field flash VEP was elicited at a constant intensity of 3.0 cd·s/m^2^ with the active electrode subcutaneously inserted in the middle of the two ears.

B, Quantitative analysis of the N1 amplitude and latency showing that A8 increased the N1 amplitude in WT but not in GPR110 KO injured mice without affecting N1 latency. No significant difference in the N1 amplitude or latency in sham animals was observed between two genotypes. The data are expressed as mean ± SEM (n=8-10). Each dot symbol represents each animal per group. ****p<0.001*, *****p<0.0001* vs. Sham-WT.

C. Average tracts of ERG at 2 weeks post injury obtained using a light-adapted (photopic) protocol.

D, E. Quantitative analysis of a and b amplitude and latency showing that A8 or injury did not change a and b amplitude and latency for both WT and GPR110 KO mice. No significant difference in these ERG parameters was observed between two genotypes.
